# Injection of Solution of Nitrate Silver into the Larynx in Membranous Croup

**Published:** 1859-03

**Authors:** J. H. Reeder

**Affiliations:** Lacon, Illinois


ARTICLE VII.

### INJECTION OF SOLUTION OF NIT. SILVER INTO THE LARYNX IN MEMBRANOUS CROUP.

BY J. H. REEDER, M.D., OF LACON, ILLINOIS.

I have long felt that we were far behind the age in the treatment of membranous croup, and especially that form which is the result of the extension of diphtheric inflammation into the air passages. While many forms of disease which, a few years ago, were considered almost necessarily fatal, have been made to yield to the advancement of medical science, membranous croup still claims a large per cent. of mortality, and the proportion of fatal cases are far too great to suit my notion of the efficacy of remedial agents.

Within the last few months, I have, in three cases, used tracheal injections of sol. nit. argentum, of from ten to forty grains of the salt to the ounce of distilled water, with the most happy results. The first case occurred in December last. H. C., daughter of the Presbyterian minister of this place, aged about six years, had a slight attack of diphtheria, which was so slight that the parents did not think it necessary to call a physician. In the course of a few days, croupy symptoms began to manifest themselves, at which time I was called to attend the case.

I found very little fever; no eruption; the tonsils were swollen and covered with diphtheric deposit, as was also the pharynx; the respiration grew especially more and more difficult, until suffocation seemed to be inevitable, notwithstanding all the means at my command were used to relieve her. I resolved to try the injection of sol. nit. silver into the trachea. I accordingly made a solution of forty grains to the ounce of water, and threw about a drachm into the trachea. The effect was astonishing. Large quantities of coagulated mucous, with fragments of detached false membrane, was immediately thrown off, with very little effort on the part of the patient, and with immediate relief of all the varying symptoms. In the course of a few hours the breathing again became labored; the injection was repeated with the same result as before. They were repeated at intervals of from ten to twenty-four hours, for three or four days, when it was no longer necessary. Very little else was done except to give, occasionally, Dover's powders, to give rest; castor oil, to move bowels once or twice daily. I have treated two cases since in the same manner, with the same result.

I used, for the purpose of giving the injections, *Warren's Laryngeal Syringe*, and think it far superior to the ordinary probang. It can be introduced with less difficulty, and the bulb being perforated laterally, directs the solution just where you want it.

